# DENV-2 Circulation and Host Preference Among Highly Anthropophilic, Outdoor-Biting *Aedes aegypti* in Dar es Salaam, Tanzania

**DOI:** 10.3390/v17060818

**Published:** 2025-06-05

**Authors:** Frank S. C. Tenywa, Silvan Hälg, Haji Makame, Jason Moore, Osward Dogan, Harubu I. Mapipi, Jane J. Machange, Nasoro S. Lilolime, Lorenz M. Hofer, Lewis D. Batao, Tunu G. Mwamlima, Pie Müller, Sarah J. Moore

**Affiliations:** 1 Vector Control Product Testing Unit, Environmental Health and Ecological Sciences Thematic Group, Ifakara Health Institute, Bagamoyo P.O. Box 74, Tanzania; hmakame970@gmail.com (H.M.); jmoore@ihi.or.tz (J.M.); oswarddogan9@gmail.com (O.D.); iddymapipi4@gmail.com (H.I.M.); jmachange@ihi.or.tz (J.J.M.); nlilolime@ihi.or.tz (N.S.L.); lbatao@ihi.or.tz (L.D.B.); tmwamlima@ihi.or.tz (T.G.M.); smoore@ihi.or.tz (S.J.M.); 2 Swiss Tropical and Public Health Institute, Kreuzstrasse 2, Allschwil, 4123 Basel, Switzerland; silvan.haelg@swisstph.ch (S.H.); lorenz.hofer@swisstph.ch (L.M.H.); pie.mueller@swisstph.ch (P.M.); 3 University of Basel, Petersplatz 1, 4001 Basel, Switzerland; 4 School of Life Sciences and Bioengineering, The Nelson Mandela African Institution of Science and Technology (NM-AIST), Arusha P.O. Box 447, Tanzania

**Keywords:** DENV, dengue fever, traps, blood feeding, longitudinal survey, serotypes, xenomonitoring

## Abstract

In Tanzania, dengue outbreaks have occurred almost annually over the past decade, with each new outbreak becoming more severe. This study investigated the prevalence of dengue virus (DENV) serotypes in the wild *Aedes aegypti* and their blood sources to determine human exposure risk in Dar es Salaam, Tanzania. A two-year longitudinal survey was conducted in the Ilala, Kinondoni, and Temeke districts of Dar es Salaam to sample *Ae. aegypti* mosquitoes using Biogents Sentinel trap (BGS), Prokopack aspiration, and Gravid Aedes trap (GAT). Collected mosquitoes were pooled in groups of 10 and tested for DENV1–4 serotypes using reverse transcription polymerase chain reaction (RT-qPCR). Blood meal sources were identified using an enzyme-linked immunosorbent assay (ELISA). Of 854 tested pools, only DENV-2 was detected and was found in all three districts: Temeke (3/371 pools), Ilala (1/206 pools), and Kinondoni (1/277 pools). Blood meal analysis showed a strong preference for humans (81%) as well as for mixed blood meals that contained human blood and other hosts (17%). Out of 354 collected hosts seeking *Ae. aegypti*, 78.5% were captured outdoors and 21.5% indoors. This study confirms the circulation of DENV-2 in *Ae. aegypti* populations, indicating a potential dengue outbreak risk in Tanzania. This study also demonstrates that xenomonitoring may be feasible in this setting. The mosquitoes’ strong preference for human hosts and predominance in outdoor settings pose challenges for dengue control efforts.

## 1. Introduction

Dengue fever is a significant global public health concern across tropical and sub-tropical regions [1]. Approximately 3.9 billion people, nearly half of the world’s population, are estimated to be at risk of infection [2]. The global incidence of dengue has risen dramatically in recent decades, with a record high occurring in 2023 [3,4] and 2024 [5]. Nearly 100 to 400 million new dengue cases occur each year [6], of which 90 million are manifested clinically, ranging from mild to severe symptoms, the latter being a life-threatening form of disease [6,7]. However, the vast majority of the cases are asymptomatic [8], leading to a likely underestimation of the true scale of the infection.

Dengue is now endemic in over 129 countries [9], with most cases occurring in Asia, which accounts for nearly two-thirds of the global burden, followed by the Americas and a small proportion of cases in the African region [10]. In Africa, dengue cases are likely to be underreported [6] due to misdiagnosis as malaria or urinary tract infection (UTI). Even when correctly clinically diagnosed, many health systems lack sufficient diagnostic capacity to detect dengue virus (DENV) [11]. Human activities contributing to climate change, globalisation, and unplanned urbanisation fuelled by rural-to-urban migration, accelerate the spread of dengue [12]. Additionally, it is predicted that with the rapid expansion of intra- and intercontinental trade, the disease is expected to spread further, potentially tripling in the next 50 years [13].

Dengue fever is caused by four antigenically distinctive virus serotypes (DENV 1–4) [14,15], which share around 65–70% genome similarity [16,17]. The virus is an enveloped, single-stranded ribonucleic acid (ssRNA) virus belonging to the Flaviviridae family and *Flavivirus* genus [18,19]. It is transmitted from a viraemic individual to another individual(s) through mosquito bites. Each serotype exhibits independent virological characteristics, where the infection by one serotype does not confer a permanent cross-immunity against the others [20]. Secondary infections with another serotype or mixed infection may potentially lead to severe forms of dengue [21,22,23]. The severity of the secondary infection is explained by the antibody-dependent enhancement (ADE) theory [24]: antibodies from a previous infection provide long-lasting immunity against the same serotype but only temporary cross-protection against others. Thus, during a subsequent infection with a different serotype, this short-lasting immunity fails to neutralise the new serotype and forms an immune complex that facilitates viral entry into host cells, enhancing virus replication and increasing disease severity. Although all four dengue virus serotypes (DENV 1–4) circulate in Africa [25,26], DENV-2 is the most prevalent [27,28,29], likely due to its greater transmissibility [30,31] and greater susceptibility among local vectors [32]. These factors have important epidemiological implications.

Dengue fever is primarily transmitted through mosquito bites, with *Aedes aegypti* and *Aedes albopictus* serving as the primary and secondary mosquito vectors, respectively [33]. Both species bite during the day, making vector control challenging, as people are active during this time, and most existing interventions offer limited protection against daytime exposure [34]. In the absence of antiviral drugs and an effective universal vaccine [35], dengue prevention and control remain dependent on vector control. Understanding *Ae. aegypti* host feeding preference and location is a critical aspect of monitoring transmission and identifying potential virus reservoirs [36]. Studies show that *Ae. aegypti* primarily feed on humans [36,37], but in the presence of alternative hosts, they may also feed on other hosts [38,39,40]. This suggests opportunistic feeding behaviour, dependent on host availability. Dengue vectors may bite indoors or outdoors [41,42,43,44], with an increased tendency to rest indoors [45] and adaptation to artificial lighting contributing to indoor biting behaviour [46].

In Tanzania, the first dengue case was reported in 1823 [25], and subsequent studies have confirmed its circulation [27,47,48,49,50,51,52]. Like other East African countries, Tanzania has recorded all four dengue serotypes [50,53,54,55], which have likely driven the frequent dengue outbreaks in the country. Reports have indicated the co-circulation of multiple virus serotypes [55,56], as seen in the 2018/2019 outbreak with DENV-1 and 3 serotypes [55]. This introduction of and co-circulation of dengue virus serotypes likely contributes to increasing disease severity. Over the past decade, dengue outbreaks in Tanzania have become increasingly frequent and severe. The most severe dengue outbreak occurred in 2019, when about 7000 cases and 13 deaths linked to DENV-1 were reported [57,58]. In 2014, over 1000 dengue cases and four deaths were recorded [59], with DENV-2 identified as the circulating serotype.

Despite frequent outbreaks recorded in recent years, dengue surveillance in Tanzania remains limited. In humans, dengue seroprevalence is mainly reported only during outbreaks [48,60,61], resulting in limited data on year-round transmission. Additionally, little information exists on the prevalence of dengue viruses in the mosquito population [62], indicating the possibility that the virus may be silently circulating in the area. Data for targeted control efforts, including *Ae. aegypti* host preference and the location (indoors or outdoors) where these mosquitoes are most likely to feed, are lacking.

Therefore, this study aimed to determine DENV prevalence in mosquitoes to assess the risk of possible dengue outbreaks and the role of xenomonitoring for low-cost and non-invasive surveillance. It also investigated the host preference and feeding location of wild *Ae. aegypti* to better understand the dengue transmission chain.

## 2. Materials and Methods

### 2.1. Study Area

**Figure 1:**
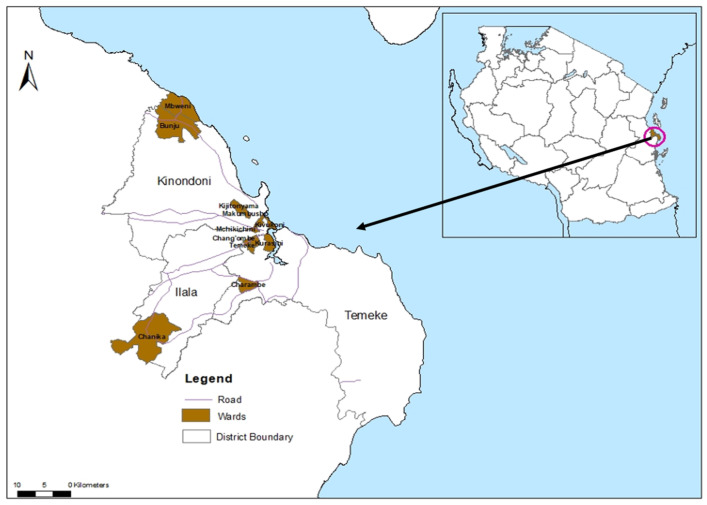
Study area where mosquito collection was conducted. Mosquito trapping was performed in three districts, with four wards selected from each district.

The study was conducted in Dar es Salaam, Tanzania’s largest economic hub (Figure 1). The city is located at 6.48′ S and 39.17′ E on the Indian Ocean coast, with a population of approximately 7 million [63]. Administratively, it consists of five districts: Ilala, Kigamboni, Kinondoni, Temeke, and Ubungo. Based on previous dengue outbreaks [53,64], Ilala (1,649,912 people), Kinondoni (982,328), and Temeke (1,346,674) [63] were selected for this study.

Dar es Salaam has a tropical climate with high temperatures throughout the year and the hottest period occurring between October to February. The city experiences one dry season between June and October and receives an average annual rainfall of 1100 mm. The short rainy season occurs from November to December, and the long rainy season extends from March to May [65].

### 2.2. Mosquito Collection

Wild adult mosquitoes were collected from June 2022 to May 2024 using Biogents Sentinel (BGS) traps (Biogents AG, Regensburg, Germany) designed for catching host-seeking *Aedes* mosquitoes, locally made Gravid *Aedes* traps (BG-GAT) [66] for sampling gravid females, and Prokopack aspirators (John W Hock Company, Gainesville, FL, USA) for collecting resting adults. Four wards were selected from each district. In each ward, 20 houses were identified, and the traps (one BGS and one GAT) were deployed in one house per day for 24 h; then, the trapped mosquitoes were collected, followed by the collection of resting mosquitoes around the premises using a prokopack aspirator. This procedure was repeated monthly per each house for 24 months. The collected mosquitoes were morphologically identified to the species level following the Wilkerson et al., 2021 identification key [67]. Female *Ae. aegypti* were pooled in groups of 10 individuals and stored in 1.5 mL Eppendorf tubes containing RNA preservation solution locally made at Swiss Tropical and Public Health (TPH) institute. The RNA preservation solution was prepared by mixing 60 mL of 0.5 M EDTA with 37.5 mL of 1 M sodium citrate in 1400 mL MilliQ water. Afterwards, 1050 g ammonium sulfate was added, and the solution was filtered through a 0.2 µm filter. Mosquitoes were analysed for blood meal and the presence of DENV.

Additionally, an experiment was conducted in six houses per month per district for three months to determine the abundance of host-seeking *Ae. aegypti* mosquitoes indoors and outdoors. A pair of BGS were deployed indoors and outdoors for 24 h, after which female mosquitoes were collected, and recorded based on their capturing location.

### 2.3. RNA Extraction and Dengue Virus Detection

#### 2.3.1. RNA Extraction

The extraction of RNA from mosquito pools was carried out using RNAzol^®^ RT (Molecular Research Center, Cincinnati, OH, USA) according to the manufacturer’s instructions. Briefly, each pool of 10 individual mosquitoes was suspended in 200 µL of RNAzol in a 1.5 mL microcentrifuge tube and manually ground using a sterile plastic pestle designated for grinding mosquitoes. The mixture was then centrifuged at 12,000× *g* for 15 min, after which the supernatant was transferred to a new 1.5 mL microcentrifuge tube. An equal volume (200 µL) of 100% isopropanol was added to precipitate the RNA, followed by incubation for 15 min and centrifugation at 12,000× *g* for 10 min. The supernatant was removed and discarded. The RNA pellet was washed twice with 200 µL of 75% ethanol, centrifuged at 4000× *g* for 3 min, and the ethanol was carefully removed. Finally, the RNA pellets were eluted with 50 µL of RNAse-free water and stored at −80 °C for molecular analysis using reverse-transcription polymerase chain reaction (RT-qPCR) [68].

#### 2.3.2. Dengue Virus Detection

A one-step multiplex RT-qPCR [69] was performed using the CFX96 Bio-Rad PCR machine (Bio-Rad Laboratories Inc., Hercules, CA, USA). The primers and probes used in the assay were adapted with modifications from Balingit et al. [70] (Table 1). The reaction was performed in 25 µL reaction volumes using the Luna^®^ Universal Probe One-Step RT-qPCR Kit (New England Biolabs, Ipswich, MA, USA) consisting of 5 µL RNA template, 10 µL of Luna Universal Probe One-Step Reaction Mix (2×), 1 µL of Luna WarmStart RT Enzyme Mix (20×), 0.8 µL each of forward and reverse primers (10 µM), and 0.4 µL of probes (10 µM). Each sample was analysed in duplicates. The RT-qPCR cycling conditions were as follows: reverse transcription at 50 °C for 30 min, initialization at 95 °C for 2 min, followed by 45 cycles of denaturation at 95 °C for 15 s, and annealing/extension at 60 °C for 1 min. RNAse-free water was used as a template for the negative control. Samples with an average cycle threshold (Ct) higher than 37 were considered negative for either DENV serotype.

### 2.4. Blood Meal Source

Blood-fed *Aedes* mosquito samples collected over two sampling years and preserved in 1.5 mL Eppendorf microcentrifuge tubes containing locally made RNA preservation solution were selected and tested for polyclonal anti-IgG antibodies targeting vertebrates commonly found in the study area, including humans, dogs, chickens, and bovines, using an enzyme-linked immunosorbent assay (ELISA) as described by Beier et al. [71]. Briefly, the abdomen of each mosquito was separated from the rest of the body parts and triturated in 1x phosphate-buffered saline (PBS) using a handheld motorised micro-pestle (DWK Life Sciences, Faust Laborbedrf AG, Schaffhausen, Switzerland). A 96-well ELISA plate (Greiner Bio-One Microlon^TM^, Monroe, NC, USA) was coated with 50 µL of Mab solution containing anti-human, anti-dog, anti-chicken, and anti-cow immunoglobulin G (IgG) antibodies produced in goat (Kirkegaard & Perry Laboratories, Gaithersburg, MD, USA) at 4 µg/mL and incubated for 30 min. After incubation, the contents were aspirated, and the excess liquid was removed by tapping the plate on a tissue paper. The wells were then filled with 250 µL of blocking buffer (BB) and incubated for 1 h. Following this, the buffer was drained, and 45 µL of BB was dispensed into each well. Next, 5 µL of each sample was loaded into the wells containing the 45 µL of BB and incubated for 2 h at room temperature. The same procedures were followed for positive and negative controls. After incubation, the plate contents were aspirated, and the wells were washed three times with 250 µL of washing buffer (PBS + Tween 20). A 50 µL aliquot of the appropriate conjugate solution (peroxidase-labelled antibody against human, dog, chicken, and cow IgG, manufactured by SeraCare Life Sciences, Milford, MA, USA) was then added to each well and incubated for 30 min at room temperature. The conjugate was removed by washing the wells four times with 250 µL of washing buffer (PBS + Tween 20). Finally, 100 µL of substrate solution (2,2′-azino-bis (3-ethylbenzothiazoline-6-sulfonic acid, ABTS)) was added to each well, followed by a 30 min incubation at room temperature. After incubation, the plates were read by observing the colour change using ELISA reader.

### 2.5. Data Analysis

All data obtained were analysed using STATA package version 16 (Stata Corp., College Station, TX, USA).

### 2.6. DENV Infection Rate in Mosquitoes

The infection rate per 1000 mosquitoes was calculated by determining the proportion of DENV-positive mosquitoes among those tested by qRT-PCR. Usually, the minimum infection rate (MIR) and maximum infection rate (MaxIR) are computed as follows:

$$MIR=\left(\frac{x}{k}\right)\times 1000$$

$$\mathrm{MaxIR}=\left(\frac{x\times m}{k}\right)\times 1000$$

However, since MIR tends to underestimate and MaxIR overestimates infection rates, both are imprecise. Therefore, the maximum likelihood estimate (MLE) with 95% confidence interval was used to provide a more accurate estimate.

$$\mathrm{MLE}=-\frac{1}{m}ln\left(1-\frac{x}{n}\right)\times 1000$$

where

*k* = total number of mosquitoes tested;

*x* = Number of positive pools;

*m* = Number of mosquitoes per pool (assuming equal pool size);

*n* = total number of pools tested.

### 2.7. Bloodmeal Preference

A descriptive analysis was performed to compare the percentage of blood-fed mosquitoes across different hosts. The anthropophagy percentage was defined as the proportion of mosquitoes with human blood meals across all districts.

### 2.8. Host Seeking Preference

A descriptive analysis was performed to compare the proportion of host-seeking mosquitoes collected indoors and outdoors. A negative binomial regression model was employed to determine if there was a statistically significant difference in host-seeking mosquitoes collected indoors versus those collected outdoors. The fixed terms in the model were location (indoors vs. outdoors), district, and ward, while day and household were included as random effects. The models estimated the mean incidence rate ratios (IRR) and 95% confidence intervals around the means.

## 3. Results

### 3.1. DENV Serotypes 1–4 Prevalence

A total of 854 pools, with 10 adult female mosquitoes per pool, were tested for DENV. Of these pools, 371 were from Temeke, 206 from Ilala and 277 from Kinondoni district (Table 2). DENV serotype 2 (DENV-2) was detected in all three districts, with Temeke having the highest maximum likelihood estimate of 0.81 per 1000 mosquitoes (Table 2). The viruses were detected in both years of mosquito sampling (Table 2), indicating endemic circulation. 

### 3.2. Host Preference

A total of 298 mosquito samples were tested for the origin of their blood meal from humans, dogs, chickens, and cows. Of these, 68.8% tested positive for either one or more blood meal sources (hosts) in the ELISA test, while 31.2% of samples showed no reaction.

**Figure 2:**
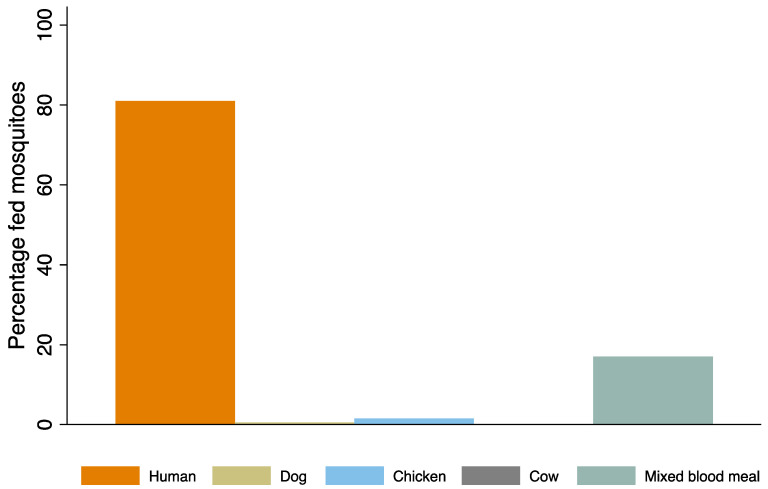
Host-feeding preference of *Aedes aegypti*. Mixed blood meal refers to mosquitoes that fed on all hosts. (Mixed blood meal = human + any of dog/chicken/cow blood meals).

*Aedes aegypti* showed a strong preference for human blood, with approximately 166 mosquitoes (81.0%) feeding on humans, followed by chicken (1.5%) and dog (0.5%) (Figure 2). About 17% of the mosquitoes had taken mixed blood meals from humans and other hosts, while none had fed on cow (Figure 2). Therefore, 98% of the mosquitoes had at least fed on humans. The majority of blood-fed mosquitoes (69.3%) were collected using the Prockopack aspirator, followed by BGS 27.8% and GAT 2.9% (Table 3).

### 3.3. Host Seeking Aedes aegypti Mosquitoes

Using BGS, a total of 354 female *Ae. aegypti* mosquitoes were collected from both indoor and outdoor locations. More than three quarters, 78.5% (*n* = 278), were caught outdoors, while 21.5% were collected indoors. The number of mosquitoes caught outdoors was 4.33 times higher than the indoor count (95% CI: [2.38–7.89], *p*-value < 0.001) (Table 4). Among the three districts, Temeke recorded a significantly higher mosquito count than Ilala (Table 4).

## 4. Discussion

Understanding pathogen circulation in vectors is crucial for disease control. This study reports the presence of DENV-2 circulating in mosquitoes from Dar es Salaam city throughout the two-year survey period, suggesting ongoing endemic virus circulation rather than a new introduction. The virus serotype reported in this study is the same as the one detected in the 2014 outbreak [53], highlighting the possibility that the virus has been persistent in the ecosystem since then. However, whole genome sequencing would be required to elucidate whether this is the case or if a new introduction of a different DENV-2 genotype occurred.

All four DENV serotypes (DENV 1–4) have circulated in Tanzania [50], with different serotypes predominating in each outbreak. This shifting pattern may explain the increasing number of dengue cases and deaths during subsequent outbreaks [58], a trend also observed in West Africa [72] and other endemic regions [4]. In 2019, WHO reported a dramatic increase in dengue cases across several countries in Africa, particularly in the sub-Saharan region [73]. This rise reflects a broader global increase in dengue in all WHO regions [6]. Despite the growing evidence, African countries, including Tanzania, lack comprehensive data on the exact magnitude of dengue virus distribution due to limited epidemiological, entomological, and virological surveillance since African vector control efforts focus mainly on malaria and dengue remains a neglected tropical disease.

Genotyping studies have shown a relatedness of dengue virus genotypes detected in East Africa, particularly Tanzania [53,59] and Kenya [74,75], to virus genotypes from Asian countries such as India and Singapore [55] as well as China [59]. This indicates that the viruses are being imported from the East via international travel and trade [59]. Africa’s rapid population growth and urbanisation will likely further accelerate the virus spread. By 2050, nearly 60% of the continent’s population is expected to live in cities [76]. Increased human mobility and urbanisation will be inevitable; therefore, deliberate dengue monitoring efforts are needed. In this context, routine screening at national and international entry points could be implemented to reduce introductions of new virus genotypes, although this may be cost prohibitive.

This study identified Temeke, Ilala, and Kinondoni districts as areas with dengue-infected mosquitoes, suggesting that these are priority areas for dengue xenomonitoring. Detection of arbovirus circulation in larval *Aedes* vectors has been conducted in 40 published studies [77] across Latin America and Southeast Asia, although this is the first study from sub-Saharan Africa to explore the potential utility of xenomonitoring for epidemic prediction. The current study evaluated DENV in adult mosquitoes using BGS, although low-cost monitoring using gravid traps is being explored [66]. The infected mosquitoes indicate a potential risk of dengue outbreaks in these areas, necessitating proactive *Aedes* mosquito surveillance. Additionally, it emphasises the need for government authorities to implement dengue control measures, including larval source reduction, targeted insecticide spraying, and public education on both mosquito control and the importance of seeking health care when experiencing dengue-like symptoms. Furthermore, it underscores the importance of implementing the International Health Regulations 2005 (IHR) to reduce the risk of virus transmission to other areas [78]. The IHR is a legal framework developed by WHO to guide the management of public health events and emergencies with the potential to cross borders. It calls on governments to undertake measures such as mapping and conducting susceptivity tests for *Aedes* vectors in urban areas, as well as implementing health surveillance at ports, airports, and borders for incoming travellers.

In Tanzania, dengue prevalence often exceeds 10% in human samples [48,56,59,60]. This study reports an average 0.04% dengue mosquito infection rate (0.41/1000), which is lower than that reported by Mboera et al. [53] during the 2014 outbreak. However, the earlier study was based on virus detection in larvae, whereas this study tested adult mosquitoes. Larval sampling may have overestimated infection rates, given they do not represent the host-seeking population, and the samples could have been biased if siblings from the same transovarially infected egg batch were sampled. Additionally, rates are likely higher due to the fact that an outbreak was ongoing. The findings from the present study align with those of Chilongola et al. [79] and Joseph et al. [80] in East Africa, Mojica et al. [81] in Nicaragua and Ecuador in Latin America, and Maneerattanasak et al. [82] in Southeast and South Asia who reported 0.3%, 2.7%, 0.7%, and 0.05–2.3% dengue infection rates, respectively, among adult mosquitoes. Similarly, this is consistent with reports on other female arthropod-transmitted diseases, such as malaria [83,84], where *Plasmodium* infection rates in *Anopheles* mosquitoes are typically very low, even in high endemic areas.

This study has demonstrated that, *Ae. aegypti* from Dar es Salaam are highly anthropophagic, more than 80% feeding only on humans and 98% overall with human blood. This behaviour significantly increases dengue transmission risk, as human–mosquito contact is a key driver of virus spread [85]. The findings of this study are consistent with research from West Africa (Senegal and Burkina Faso) [41,86,87] as well as India [39] South East Asia (Thailand) [36], Australia [88], Latin America (Brazil, Ecuador, and Peru) [89], and North America (USA) [42], which all indicated high anthropophagy among *Ae. aegypti*.

*Aedes aegypti aegypti* (*Aaa*) and *Aedes aegypti formosus* (*Aaf*) are the *Ae. aegypti* subspecies commonly found in Africa [90]. *Aaa* is considered an urban mosquito primarily responsible for urban dengue and yellow fever transmission [91]. *Aaf* inhabits peri-urban environments and serves as an agent for sylvatic dengue as well as yellow fever transmission [91,92] and is less competent for dengue [93] and less anthropophilic [94]. In this study, we used morphological identification only and were unable to distinguish between the two subspecies. Given the lack of comprehensive research on their coexistence in the country, our findings emphasise the need for genomic studies to accurately characterise them.

As reported in other studies [40,41,86], human–animal mixed blood meals were frequently observed in this study, highlighting the feeding flexibility of *Ae. aegypti*. This mosquito species is known for transmitting dengue virus and other viruses, including yellow fever virus (YFV), Chikungunya virus (CHIKV), and Zika virus (ZIKV), all of which pose a substantial public health burden. While the ability of *Ae. aegypti* mosquitoes to feed on multiple animal species has implications for virus transmission, such as cross-species transmission and vector competence; the role of animals as reservoirs for DENV, CHIKV, YFV, and ZIKV in urban settings remains unlikely. Studies have described systems demonstrating the potential co-infection of DENV serotypes in an individual [95,96]. With all four DENV serotypes circulating in the country [50], it is likely that the mosquitoes’ ability to feed on multiple human hosts could essentially lead to individuals being infected with more than one virus serotype; this will potentially increase the chance of individual developing severe dengue if he/she was previously infected with another virus serotype.

Moreover, the locations where mosquitoes seek blood meals from hosts and rest after feeding have important implications for vector control strategies. This study demonstrated that more than three quarters of *Ae. aegypti* preferentially seek blood meals outdoors, presenting a challenge for control. Most mosquito control interventions used in Tanzania are designed for malaria control and are applied indoors, targeting indoor host-seeking and resting mosquitoes. This finding is similar to studies of *Ae. aegypti* in Burkina Faso [87,97,98] and Ghana [99] that also indicate outdoor feeding. Consequently, larval source reduction [100] might be the most likely intervention to be successful at the community level. Further work is ongoing to measure the resting behaviour and susceptibility of the Dar es Salaam population of *Ae. aegypti* to insecticides used for mosquito control. In addition, the use of *Wolbachia*, an endosymbiotic alpha-proteobacterium naturally occurring in arthropods, has shown excellent efficacy in preventing dengue when deployed at a city scale [101]. *Wolbachia* blocks dengue virus replication, thereby reducing the ability of mosquitoes to transmit the disease. Its introduction also induces cytoplasmic incompatibility (CPI), whereby uninfected females that mate with *Wolbachia*-infected males cannot produce viable offspring. Because Wolbachia is maternally inherited, the trait spreads through the mosquito population over time, rendering it refractory to DENV. This intervention is self-sustaining and may be particularly valuable in rapidly growing urban areas like Dar es Salaam.

### Study Limitations

To fully understand the indoor and outdoor ecology of *Ae. aegypti*, traps targeting host-seeking, resting, and oviposition behaviours need to be deployed concurrently both indoors and outdoors. However, we were unable to collect indoor mosquitoes using GATs due to the unpleasant smell of the infusion and with prokopack aspirators because of restricted access to homes, usually because residents were absent. Consequently, indoor collections were limited to BGS, a trap type designated for collecting host-seeking mosquitoes. Therefore, we recommend that for future studies, the indoor and outdoor mosquito collection should also include resting collections because the use of traps with a lure may bias the collections towards human-fed mosquitoes [89]. Additionally, blood meal analysis for host preference was performed on only four hosts (humans, dogs, chickens, and cows). However, we found that 93 samples did not react suggesting that the mosquitoes may have contained blood meals from hosts not included in the analysis. In Kenya, *Ae. aegypti* has been found to feed on goats, rats, and cats [101], which were also present in the present study site but not tested for. Therefore, future studies should include a broader range of potential hosts.

## 5. Conclusions

This study confirms the circulation of DENV-2 in the mosquito population in Dar es Salaam, highlighting the risk of a potential dengue outbreak in Tanzania. Dar es Salaam is one of Africa’s major metropolitan cities, with a population of nearly seven million. It serves as the economic hub of Tanzania, so it experiences a significant influx of local and international travellers. The presence of DENV-2 in mosquitoes year-round, as well as the strong human feeding preference of *Ae. aegypti* indicates the potential risk of DENV transmission to humans. These findings emphasise the need for enhanced surveillance and targeted proactive vector control measures, including the removal of breeding sites to mitigate dengue outbreaks.

## Figures and Tables

**Table 1:** Primers and Probes used for DENV serotyping from *Aedes aegypti* mosquito samples. The primers and probes were adapted as per Balingit et al. [70] modification.

DENV Serotype Detected	Primer and Probes	Nucleotide Sequence (5′ → 3′)	Fluorophore and 3′ Quencher
DENV-1	DEN-1 forward	CAAAAGGAAGTCGTGCAATA	FAM
	DEN-1 reverse	CTGAGTGAATTCTCTCTACTGAACC	
	DEN-1 probe	CATGTGGTTGGGAGCACGC	
DENV-2	DEN-2 forward	CAGGCTATGGCACTGTCAC	HEX
	DEN-2 reverse	CCATTTGCAGCAACACCATC	
	DEN-2 probe	CTCTCCGAGAACGGGCCTCGACTTCAA	
DENV-3	DEN-3 forward	GGACTGGACACACGCACTCA	CY5
	DEN-3 reverse	CATGTCTCTACCTTCTCGACTTGTCT	
	DEN-3 probe	ACCTGGATGTCGGCTGAAGGAGCTTG	
DENV-4	DEN-4 forward	TTGTCCTAATGATGCTGGTCG	CY5.5 CY5/BHQ3
	DEN-4 reverse	TCCACCTGAGACTCCTTCCA	
	DEN-4 probe	TTCCTACTCCTACGCATCGCATTCCG	

**Table 2:** DENV serotype detected from pooled *Aedes aegypti* mosquitoes in Dar es Salaam.

Districts	Mosquito Samples				
	Pools Tested	Positive	DENV-Serotype	Detection Year	Infection Rate per 1000 Mosquitoes
Temeke	371	3	DENV-2	2023 and 2024	0.81 (0.18, 2.39)
Ilala	206	1	DENV-2	2024	0.49 (0.012, 2.80)
Kinondoni	277	1	DENV-2	2023	0.39 (0.009, 2.20)
Total	854	5	DENV-2	2023 and 2024	0.41 (0.013, 1.10)

**Table 3:** Blood-fed *Aedes aegypti* mosquitoes collected by trap type.

Traps	BGS	Prokopack Aspirator	GAT	Total
Blood fed *Aedes aegypti*	57	142	6	205
Percentage blood fed	27.8	69.3	2.9	100

**Table 4:** Percentage and incidence rate ratio (IRR) of host-seeking *Aedes aegypti* collected indoors and outdoors.

	N	*n* (%)	IRR (95% CI)	*p*-Value
Collection location				
Indoors	54	76 (21.5)	1	-
Outdoors	54	278 (78.5)	4.33 (2.38–7.89)	<0.001
Districts				
Ilala	18	42 (11.9)	1	-
Kinondoni	18	135 (38.1)	4.24 (1.98–9.06)	<0.001
Temeke	18	177 (50.0)	5.03 (2.39–10.58)	<0.001

Legend: N = collection days, *n* = number of mosquitoes, IRR = incidence rate ratio, 95% CI = 95% confidence interval.

## Data Availability

The datasets generated during the study are available from Ifakara Health Institute and the corresponding author upon reasonable request.

## Abbreviations

BGS	Biogent sentinel trap
GAT	Gravid *Aedes* trap
DENV	Dengue virus
ITN	Insecticide-treated net
IRS	Indoor residual spray
DF	Dengue fever
YF	Yellow fever
CHIK	Chikungunya
ZIK	Zika
IHR	International health regulations
WHO	World health organisation
IRR	Incidence rate ratio
OR	Odds ratio
ELISA	Enzyme-linked immunosorbent assay.
qRT-PCR	Quantitative reverse transcriptase polymerase chain reaction
MIR	Mosquito infection rate
